# Mothers’ eating disorder history and mother and infant attention to food during infant meal times: a candidate for intergenerational transmission of eating disorder behaviours

**DOI:** 10.3389/frcha.2025.1699643

**Published:** 2026-01-16

**Authors:** Fay Huntley, Rebecca M. Pearson, Ilaria Costantini, Marc H. Bornstein, Amy Campbell, Miguel Cordero, Nicky Wright

**Affiliations:** 1 School of Health in Social Science, Clinical Psychology, University of Edinburgh, Scotland, United Kingdom; 2 School of Psychology, Faculty of Health and Education, Manchester Metropolitan University, Manchester, United Kingdom; 3 Population Health Sciences, University of Bristol Medical School, Bristol, United Kingdom; 4 Division of Psychiatry, University College London, London, United Kingdom; 5 Eunice Kennedy Shriver National Institute of Child Health and Human Development, Bethesda, MD, United States; 6 Institute for Fiscal Studies, London, United Kingdom; 7 MRC Integrative Epidemiology Unit, University of Bristol, Bristol, United Kingdom; 8 School of Psychological Science, University of Bristol, Bristol, United Kingdom; 9 Centre for Academic Mental Health, University of Bristol, Bristol, United Kingdom; 10 Instituto de Ciencias e Innovación en Medicina, Universidad del Desarrollo Facultad de Medicina Clínica Alemana, Las Condes, Chile

**Keywords:** mothers, infants, eating disorder, attention, food, interactions

## Abstract

**Introduction:**

There is evidence to suggest that individuals with eating disorders (EDs) show differences in attention to food compared to those without eating disorders. Children of mothers with eating disorders are at an elevated risk of developing an eating disorder themselves. One potential intergenerational pathway may be that parents, and then infants, pay more attention to food in interactions, which in turn mediates transmission of disordered eating behaviours, particularly those during feeding and mealtimes. No study has investigated whether mothers’ ED behaviour history is associated with maternal and infant attention to food during infant feeding interactions.

**Methods:**

Mothers and 7-month-old infants from the Avon Longitudinal Study of Parents and Children Generation-2 provided video footage of mother–infant feeding interactions filmed at home using head cameras. Feeding interactions were coded for mothers’ and infants’ visual attention using a micro-behavioural observational coding system. Outcomes were the mothers’ and infants’ proportion duration (of the entire feeding interaction) spent looking at food. Mothers’ ED behaviour history was assessed at age 25 years using the Youth Risk Behaviour Surveillance System questionnaire, from which a binary Diagnostic and Statistical Manual of Mental Disorders, fifth edition, disordered eating variable was generated. Linear regression testing associations between mothers’ ED behaviour history and mother and infant attention to food were adjusted for infants’ sex, age, and birth order, and mothers’ age, education, and employment.

**Results:**

In 98 mother–infant dyads, mothers’ ED behaviour history was associated with longer proportion duration of infants’ looking at food during mealtimes [estimate = 0.59 (0.19, 0.99), *p* = 0.004], corresponding to a 20% difference in time spent looking at food for infants of mothers with an ED behaviour history compared to those without. There was no association between ED behaviour history and mothers’ attention to food.

**Conclusions:**

Increased infant looking time to food during feeding may indicate a preoccupation with food. This preoccupation could be a reflection of ED behaviours being modelled to offspring by mothers or early behavioural markers of shared genetic risk for EDs. Support for mother–infant dyads with an ED history could target guiding mothers’ and infants’ attention (via video feedback, for example) to non-food-related activities to minimise any impact on the mothers’ relationship with the infant or the infants’ relationship with food.

**Lay summary:**

Children of mothers with EDs are at an elevated risk of developing an eating disorder themselves. One potential pathway of transmission of eating disordered behaviour from mother to offspring may be through behaviours shown during feeding and mealtimes. Existing evidence suggests that individuals with eating disorders show differences in attention to food compared to those without eating disorders, but no study had examined attention to food in mother-infant interactions. Using naturalistic observations of feeding at home, we showed that the infants of mothers with an eating disorder history showed increased attention to food during mealtimes. This provides preliminary evidence that attentional patterns during feeding interactions could be sensible targets for intervention.

## 1 Introduction

Eating disorders (EDs) are characterised by severe disruptions in eating and other weight/body image-related behaviours that significantly impact an individual’s emotional and physical wellbeing and psychosocial functioning (1, 2). Children of mothers with EDs are at an elevated risk of developing EDs themselves (3). Pathways of transmission include shared genetic influences and environmental factors, including childhood and early adolescent exposures, sociodemographic influences, and body image difficulties (4, 5). An unexplored pathway is through early behavioural responses to food in parent–infant interactions. There is evidence that concerns around infant feeding are widely reported by mothers. In the present study, we utilise a multigenerational cohort study to explore ED behaviour history in mothers and early observed interactions during feeding at 7 months and test the hypothesis that early mother and infant attention to food is different in mothers with ED history.

Environmental factors are of high importance, given that they may be amenable to intervention and modification to reduce risk (2, 6). Infants’ early years are an important time in the development of feeding behaviours and regulatory mechanisms relating to internal and external cues of hunger, satiety, and emotional states (7, 8). Mealtimes and other feeding interactions represent key interaction periods between mothers and infants (9, 10). These dyadic interactions could constitute a candidate process for the relationship between mothers’ EDs and infants’ eating/food-related difficulties (11, 12). The modelling of observable attitudes, comments, and/or specific behaviours related to mothers’ eating difficulties may result in the transmission of disordered eating behaviours to their infants (11, 13).

Some existing evidence has examined associations between maternal EDs and self-reported infant feeding interactions and practices. In the Avon Longitudinal Study of Parents and Children (ALSPAC), mothers with anorexia nervosa reported more difficulties in five out of six feeding difficulty domains, including exhaustion with feeding, slow feeding, child not satisfied or hungry after feeding, and lack of established feeding routine. Mothers with bulimia nervosa reported more refusal from their infants to take solid food than those with anorexia nervosa, other psychiatric disorder/s, or no disorder. Mothers with other psychiatric conditions reported more feeding difficulties compared to those without any disorder (11). In the same sample, Micali et al. (14) found that mothers’ lifetime history of any ED symptoms was associated with feeding difficulties at infant ages 1 and 6 months. Mothers’ ED symptoms in pregnancy also predicted feeding difficulties at 1 month postpartum. In testing direct and mediational models, Micali et al. (14) found that mothers’ lifetime history of ED symptoms predicted infant feeding difficulties both directly and mediated by perinatal maternal anxiety and depression symptoms. In a prospective panel study of toddlers to preschool-aged children, mothers who reported a history of binge EDs were significantly more likely to report using unsupportive distress-focused responses during feeding interactions. These responses are characterised by mothers’ reactions to infants’ emotions that are focused on their own dysregulation, rather than alleviating their infants’ emotions. Maternal binge EDs were prospectively associated with more non-responsive feeding practices, including using certain foods (e.g., follow-up sweet treats) as a reward for eating, persuasive/pressured eating, and over-restriction of food at 2-year follow-up (15).

The above studies used maternal reports about their own psychopathology and their infants’ behaviours, which are subject to social desirability and/or reporter bias, mood state biases, and shared method variance (16). Several observational studies have used the Chatoor Feeding Scale [CFS, (17)], a global coding system for observations with the following five subscales: dyadic reciprocity, dyadic conflict, talk and distraction, struggle for control, and maternal non-contingency. Squires et al. (18) conducted a small-scale observational study of mother–child dyads recruited from first obstetric consultations, with mother–infant interactions observed at home at 8 weeks postpartum. They compared mothers who met criteria for lifetime EDs based on a cut-off score on the Eating Disorder Examination questionnaire to those who did not. The mothers’ ED scores were negatively correlated with dyadic reciprocity, which reflects the quality of relatedness and affective engagement between the mother and the infant during mealtimes. When compared to the control group, mothers with EDs were less engaged in dyadic and sensitive interactions during mealtimes.

In a clinical sample, Cimino et al. (19) longitudinally investigated the influence of a binge ED diagnosis, in one or both parents, on parent–infant feeding interactions at 18 months postpartum (204 mother/father–child dyads) using the Italian version of the CFS. Parent–child dyads in the binge ED group, compared with a control group, showed poorer feeding interactions on all CFS subscales. In follow-up, Cimino et al. (20) found that the observed feeding difficulties at 18 months mediated the association between the mothers’ ED symptoms at 18 months and child anxiety and affective problems at 36 months. Another study compared 31 mothers within a prospective birth cohort who met criteria for three ED diagnoses to 20 control mothers on the CFS codes during a mealtime observation at 10 months (8). The mother–child dyads in the ED group scored significantly *lower* on the struggle for control subscale compared to the dyads in the control group. There were no significant differences between the two groups for the other subscales. The authors suggested the finding may be explained by the mothers overcompensating for their urge for control during feeding due to concerns about passing on behaviours related to their ED.

In summary, there is some, not entirely consistent, observational evidence that mothers with EDs (current or history) show differences in infant feeding interactions compared to mothers without EDs. Existing evidence has used global coding schemes to characterise behaviour during feeding that rely on coders’ interpretations, typically combined across multiple different units of behaviour (e.g., speech, tone of voice, and eye gaze), which precludes an examination of specific behaviours. Another key limitation is the focus on mothers’ behaviour only. A full test of an intergenerational pathway would include changes not just in the mother but in the infant’s attention to food. Uniquely, we also explore whether infants look more at food in their first interactions with food at 6 months.

One relevant area of interest is differences in attentional processes. Attentional allocation controls which aspects of our environments we assign further cognitive effort to. Individual differences in attentional processes, often referred to as biases, are associated with clinically relevant behaviours (21). Pearson et al. (21) found that mothers’ depressive symptoms in late pregnancy were associated with an attentional bias towards distressed infants’ faces. In relation to feeding behaviours and interactions, attentional biases towards food in mothers and/or infants may index a preoccupation with food. Some evidence using experimental attentional and eye tracking paradigms points to differences in attentional processes in individuals with EDs in relation to food (22–24). Individuals with EDs have been found to show more attentional bias and faster visual orientation to food (22–28), although some studies suggest an avoidance of food stimuli (29) or no associations (30). Inconsistencies in the literature may be related to disorder-specific mechanisms (i.e., differences between subtypes of EDs). There is also variation in conceptualisations of eating behaviours, the experimental paradigms used (inferring attention vs. measuring focus of attention directly), and variation in sample characteristics (30).

One way of examining attentional processes is through gaze. For mothers and infants, gaze is a key non-verbal cue during interactions, signalling intention/s or emotional states, initiating joint attention, and making requests (31). Feeding interactions involve a range of communicative strategies to signal appetite and satiety (9, 32), and infant gaze during feeding is thought to serve as a non-verbal indicator of hunger or satiation (9, 31). Observational studies have examined infant gaze patterns during feeding and show that gaze at food is usually higher during the initial stages of feeding and that infants move flexibly between gazing at food and gazing at the caregiver, with mutual gaze also playing an important role in feeding interactions (31, 33). For a responsive, bi-directional process, the caregiver’s role is to recognise and appropriately respond to infant cues so that feeding meets the infant’s needs. The mother’s gaze also serves to cue the infant (33). Given evidence indicating that individuals with ED may show biases in attention to food, this may disrupt the dyadic function of gaze during feeding interactions. A maternal bias towards food, reflecting a pre-occupation with food, could undermine the flexible, responsive patterns required for effective feeding interactions. In turn, infants of mothers with an ED history may show increased bias to food by responding to mothers’ cueing gaze to food or by increasing their use of gaze to index hunger, as the mothers may be less responsive if their attention is directed towards food.

No studies to date have examined mothers’ and infants’ attention to food and possible attentional biases in naturalistic scenarios, i.e., participants’ homes. Neither have they investigated whether mothers’ ED behaviour history is associated with *both* the mother’s and infant’s attention to food. To do this, micro-coding of maternal and infant gaze is needed during feeding interactions. Mother–infant observations are typically conducted in laboratory environments/clinic settings with structured tasks or in researcher-administered home visits. Such methods are likely to lead to reactivity effects, where participants change behaviour/s due to the presence of the researcher or knowledge that they are being observed or recorded (34). Evidence for the improved ecological validity of observational assessments conducted using wearable cameras with infants and their families informed the current study, which was designed to overcome the above shortfalls. This method has the advantages of capturing a first-person perspective on interactions of interest, improving capture of faces and eye gaze, and recording unstructured tasks in the home, such as feeding and mealtimes (35, 36).

The present study aimed to explore the association between a maternal history of ED behaviours and increased maternal and infant gaze at food during infant feeding, using data from a large prospective birth cohort. Mother–infant feeding observations were captured using head cameras in participants’ homes during unstructured tasks when the infant was 6 months old. Video records of observations were micro-coded for mother and infant visual attention and mothers’ facial expression. We hypothesised that both the mothers and the infants of mothers with an ED behaviour history would show a longer duration of looking at food compared to mothers without an ED behaviour history.

## 2 Materials and methods

### 2.1 Participants

The sample comprised participants from the ALSPAC cohort. In the original recruitment, 14,541 pregnant mothers residing in South West England were recruited (ALSPAC-G0) from 1991 to 1992. This sample included 15,454 pregnancies and 14,901 babies (ALSPAC-G1) were alive at 1 year of age. In 2012, the recruitment of members of ALSPAC-G1 and their partners, who had their own children, into ALSPAC-G2 began. Data were collected from both parents (at least one of whom was a G1 participant) and their children. Ethical approval for the study was obtained from the ALSPAC Law and Ethics Committee and the Local Research Ethics Committees. Recruitment of mothers into the head camera study began on 7th July 2016. Data collection was halted due to COVID-19-related measures from March 2020 to April 2021, when virtual visits were introduced. From January 2022, face-to-face clinic visits were reintroduced. Altogether, 439 mothers were invited to participate in the head camera study, of whom 241 (55%) accepted the invitation and 155 (35%) agreed to record their interactions with their infant using the head cameras at home. This analysis focuses on 98 mother–infant dyads who provided observations of feeding and data on ED behaviour history. Compared to the full sample, these 98 mothers were younger (mean age = 27.15 years, SD = 1.22, vs. full sample mean age = 28.39 years, SD = .49), more likely to have female infants (65.30% female vs. 48.8% in the full sample), and more educated (38.77% with a degree or higher education vs. 22.94% in the full sample).

Further details on the cohort profile, representativeness, and phases of recruitment, including ALSPAC-G2, are described in Boyd et al. (37) and Fraser et al. (38), with further updates in Lawlor et al. (39) and Northstone et al. (40). The ALSPAC study website (https://www.bristol.ac.uk/alspac/) contains details of all the data that are available through a fully searchable data dictionary and variable search tool (http://variables.alspac.bris.ac.uk/).

### 2.2 Ethical approval

Informed consent for the use of data collected via questionnaires and clinics was obtained from participants following the recommendations of the ALSPAC Ethics and Law Committee at the time of data collection.

### 2.3 Procedure

#### 2.3.1 Videorecording procedures using the head cameras

Mother–infant interactions were captured at home using head cameras worn by both the mother and infant, which capture a front-on face view from the mother's and infant’s perspectives. Head cameras are reliable for capturing mother and infant behaviours (36). The parents were given head cameras and asked to use them at home during mealtime and play interactions; the mealtime interactions were used for this study.

#### 2.3.2 Exposures

##### 2.3.2.1 Maternal ED behaviour history

Mothers’ ED behaviour history was measured using the Youth Risk Behaviour Surveillance System questionnaire (41) at the age of 25 from the ALSPAC assessment. We used questions about behaviours in the previous year to lose weight or avoid gaining weight: (1) fasting for at least a day; (2) purging (vomiting or taking laxatives/other medications); (3) excessive exercise (exercise that frequently interfered with daily routine/work or frequently exercising even when sick/injured); and (4) binge-eating with a sense of loss of control. The primary exposure of interest was any disordered eating, a composite measure derived from any report of fasting, purging, excessive exercise, or binge-eating at any frequency. We did not make a diagnosis of an ED and did not incorporate any other diagnostic criteria into the variable. Of the 98 mothers in this sample, 22 (22.4%) had endorsed at least one item, which we used to index a history of ED behaviours. This is comparable to a prior publication reporting on the entire ALSPAC sample (42).

#### 2.3.3 Outcomes

##### 2.3.3.1 Behavioural assessment of mother–infant interactions: facial expressions and visual attention behaviours

The footage from the interactions was coded using the event-based Mental Health Intergenerational Transmission (MHINT) micro-coding scheme (43) and in Noldus Observer XT® 16.0 software (44). In event-based coding, once a behaviour is coded, for example, when a parent looks at an object, they will be coded as looking at the object until that changes; that is, codes are updated according to new “events.” Within each behavioural group (e.g., caregiver visual attention), the specific behaviours (e.g., looking at the infant or looking at the focus object) are mutually exclusive. For more details on the coding of visual attention, see Costantini et al. (44). The MHINT manual includes multiple different behaviours; visual attention to food is used in this study. The outcome variables represented the proportion of time during the feeding interaction in which attention to food by the mother or infant was observed. The coding was performed by five coders who were postgraduate level or above. The coders were trained on three initial videos by the core coder. Every one in five videos was double-coded. Weekly review consensus sessions were held in the first 6 months of coding, where any coding discrepancies were discussed and resolved. Inter-rater reliability was assessed through double-coding of a subset of videos. Agreement between coders was evaluated with Cohen's kappa (*κ*) and was calculated separately for each behavioural group. Reliability was generally high, with *κ* values ranging from 0.67 to 0.85 for facial expressions to 0.75–0.84 for visual attention in the mothers, and exceeding 0.90 for several other groups [see Table 1 in Burgess et al. (45)].

**Table 1:** Mean and SD of the proportional durations of infant and mother looking at food.

Variable	Proportional duration of the infant looking at food		Proportional duration of the mother looking at food	
	*N*	Mean (SD)	*N*	Mean (SD)
ED history	22	0.93 (0.70)	22	0.47 (0.98)
No ED history	76	0.28 (0.83)	76	0.43 (1.62)

#### 2.3.4 Covariates

Infant sex recorded at birth was coded as 1 = female and 2 = male, with other covariates including birth order (1 = first born, 2 = second or third born), infant age in months, mothers’ age in years, education (1 = undergrad degree or higher, 0 = less than undergrad degree), and mothers’ employment (1 = employed, 0 = unemployed).

### 2.4 Statistical analyses

Statistical analyses were conducted using Stata 17 (46). Linear regression was used to predict the proportion duration of attention to food from the binary mothers’ ED behaviour history variable. A one-unit increase in proportion attention corresponded to approximately a 40-percentage point increase in raw behaviour duration. Given that most observations lasted 6 min (300 s), this would equate to a difference between mothers with ED behaviour history and mothers without ED history of approximately 120 s. Accordingly, a coefficient of 0.25 would imply a 10-percentage point (or 30-s) difference in the duration of a given behaviour (43).

We used a directed acyclic graph to identify relevant confounding variables (Supplementary Figure S1). Maternal education and employment were also identified as relevant confounding variables. However, missing data on these variables reduced the overall sample and reduced the numbers with ED behaviour history to 15, which also contributed to the limited variability in maternal education in those with ED behaviour history. Thus, secondary sensitivity-adjusted models were estimated that included maternal education and employment.

We conducted a Monte Carlo simulation-based power analysis in Stata 19.5 (47) to estimate whether we had sufficient power to detect a medium to large effect in a linear regression with a binary predictor (group sizes: 22 vs. 76). We used an expected standardised slope of 0.86, corresponding approximately to a medium-to-large effect (f² ≈ 0.15–0.35) (48), and used the observed standard deviation of the outcome variable. For each sample size, we ran 1,000 simulations, estimating the power as the proportion of models in which the predictor was significant at *p* < 0.05. The results indicated that a sample size of 98 would achieve ≥80% power to detect a medium to large effect.

## 3 Results

Descriptive statistics are presented in Table 2. Mean maternal age for mothers with an ED history was 26.14 years and their infants’ mean age was 7.41 months. The infants of the mothers with an ED history were more likely to be female and not first born.

**Table 2:** Sample characteristics reported separately for mothers with or without ED behaviour history.

Variable	ED history (*N* = 22)	No ED history (*N* = 76)
	Mean (SD)	Mean (SD)
Maternal age (years)	26.14 (1.25)	27.45 (2.10)
Infant age (months)	7.41 (0.50)	7.13 (1.05)
	*N* (%)	*N* (%)
Mother employment		
Full-time/part-time	15 (68.18%)	58 (76.32%)
Mother education		
Undergraduate degree or higher	<5 (<5%)^a^	38 (50.00%)
Child gender		
Female	16 (72.73%)	48 (63.16%)
Male	6 (27.27%)	28 (36.84%)
Birth order		
First	13 (59.09%)	56 (73.68)
Second or third	9 (40.91%)	16 (26.31%)

a This may include zero.

There was no association between ED history and the mothers’ looking at food (*p* = 0.329 after adjusting for infant sex, age, birth order, and mother’s age). Of the covariates, increased infant age and decreased maternal age were associated with the mother spending more time looking at food. However, mothers’ ED behaviour history was associated with increased infant time spent looking at food [estimate = 0.59 (95% CI 0.19, 0.99), *p* = 0.004], after adjusting for infant sex, age, birth order, and mother’s age. The coefficient of 0.59 equated to an approximately 60 s difference in infant looking time at food for infants of mothers with ED behaviour history compared to those without. Of the covariates, increased infant age was significantly associated with proportionally longer looking at food, and female gender was marginally associated with longer looking.

We then ran an additional model including mothers’ education and employment as covariates, which reduced the sample to 15 cases with ED behaviour history and 74 without ED history. ED behaviour history still predicted increased infant looking at food [estimate = 0.53 (95% CI 0.01, 1.04), *p* = 0.004], and maternal education was significantly associated with increased infant looking at food [estimate = 0.55 (0.07, 1.03), *p* = 0.025]. Table 1 shows the means and SDs for the proportion duration of looking at food in mothers with and without an ED behaviour history. Table 3 shows the results of the linear regression models predicting the proportion duration of looking at food during feeding from the ED behaviour history.

**Table 3:** Adjusted linear regression models predicting the proportional duration of episodes of looking at food for the mothers and infants.

Variable	Proportion duration of the mother looking at food		Proportion duration of the infant looking at food	
	Estimate [95% CI]	*p*	Estimate [95% CI]	*p*
Infant sex	0.03 [–0.45, 0.50]	0.917	0.32 [–0.01, 0.66]	0.057
Infant age	0.56 [0.29, 0.82]	<0.001	0.20 [0.02, 0.38]	0.032
Birth order	0.03 [–0.40, 0.46]	0.882	0.04 [–0.25, 0.32]	0.786
Mother age	–0.14 [–0.27, –0.01]	0.031	0.02 [–0.07, 0.11]	0.603
ED history	–0.27 [–0.85, 0.29]	0.329	0.59 [0.19, 0.99]	0.004

In this study, as in prior ALSPAC publications, we used a binary variable reflecting the presence of any eating disordered behaviours, with eating disorders being those outlined in the Diagnostic and Statistical Manual of Mental Disorders (DSM). However, we also plotted the association between a count of ED behaviours and mother and infant attention to food to allow us to visualise the associations, which are shown in Supplementary Figures S2, S3. Consistent with the regression analyses, there was a linear relationship between ED history and infant attention to food, and no evidence of an association with mothers’ attention to food.

## 4 Discussion

This was the first study to examine mother and infant attention to food during naturalistic feeding interactions. We hypothesised that mothers and infants of mothers with an ED behaviour history would spend proportionally more time looking at food compared to those without an ED behaviour history. Consistent with our hypothesis, we found that infants of mothers with an ED behaviour history showed greater duration of attention to food, after accounting for infant sex, birth order, maternal age, education, and employment status. There was no association between mothers’ ED behaviour history and mothers’ attention to food.

The existing evidence on ED history and mother–infant interaction is inconsistent, but several studies have suggested difficulties in mothers’ behaviour during feeding (8, 11–13). We focused specifically on attention to food during feeding interactions, which had not previously been examined, with the hypothesis that increased attention to food may index an attentional bias, i.e., towards food. Whilst mothers showed no differences in attention to food, infants of mothers with ED behaviour history showed longer duration of looking at food, with the difference equivalent to 20% or 60 s in a 300 s interaction.

We drew on experimental evidence showing that individuals with a history of EDs show differences in attention to food stimuli to develop the hypothesis that mothers with ED history may show a greater preoccupation with food during feeding interactions with their infants. Attention to food has also been examined in developmental studies of feeding interactions, which identify the role of attention to food and flexible shifting of attention between food and mutual gaze as serving as infant cues for hunger and satiety in responsive feeding interactions. In this context, maternal attentional bias to food could affect infant gaze, either via cueing infant attention to food or via reducing her overall responsiveness, leading the infant to change their gaze patterns to communicate. In this study, we did not observe any differences in maternal gaze to food, but infants of mothers with an ED history showed increased attention to food. There are multiple possible explanations for this pattern of findings. As suggested by Doersam et al. (8), mothers with an ED history may change their behaviours or overcompensate during feeding interactions with their infant. It is possible that maternal attention to food is modelled to infants whilst mothers themselves are eating, rather than occurring during mother–infant feeding interactions (51). It may also be possible that there are other differences in verbal and non-verbal maternal behaviours during mother–infant feeding interactions that impact infant attention to food that were not measured in this study. The present findings could reflect that infants’ behaviour is driven by shared genetic liability for EDs, consistent with evidence that EDs are moderately heritable (5, 49), rather than mothers’ behaviour during feeding interactions. It may be that infants with a propensity to develop EDs experience difficulties or differences in identifying their own internal cues of hunger or satiety, contributing to the increased attention to food observed in these infants. Attentional bias towards food-related stimuli may set the conditions for overevaluation/preoccupation with food to develop for these infants, particularly given the importance of the first years of life for feeding behaviours, regulatory mechanisms and understanding about food (9, 10). An increased responsiveness to food reported by parents in a questionnaire has been linked to later child weight outcomes (50), although no studies have examined ED outcomes. Future research is required to examine the long-term outcomes of increased infant attention to food.

The strengths of this study include that it is the first to examine attention to food in mother–infant feeding dyads using observations of at-home naturalistic interactions without the presence of an observer. The use of head cameras increases the ecological validity of the findings, as compared to observations carried out in laboratory settings or at home with the presence of an observer (36). Using this observational technique allows the capture of attentional patterns and behaviours that may be less likely to occur when in the presence of an observer or in an artificial setting (35, 36).

Several limitations should be noted. The findings should be interpreted with caution due to the small sample size. Our study was conducted with a general population sample of mothers who reported on the presence of any ED behaviours. This was not a sample of those who would have met the diagnostic criteria for an eating disorder diagnosis. The results, therefore, may not apply to mothers who meet the clinical thresholds for eating disorder diagnoses. Further, in this analysis, all forms of EDs were combined because the sample of mothers with ED behaviour history was small (*N* = 22). Only a small number of questions were asked in the ALSPAC cohort, with only one item indexing restriction (fasting for 1 day, which may mean that other forms of dietary restriction are not well represented. There is some evidence that different ED presentations (i.e., anorexia, bulimia, or binge eating disorder) may be associated with different attentional processes and different correlates with parent behaviours during feeding interactions. Exploring such potential variations is an important area for future exploration.

We investigated attention to food during infant feeding interactions, but the mother's attention to food during her own mealtimes is also likely an important context for modelling attention to food. Future research should collect observations of both mothers’ and infants’ mealtimes. Additionally, we were unable to examine some potentially relevant variables, such as whether the infants in our sample had been breastfed and the technique of feeding used by mothers (e.g., spoon-fed or baby-led weaning). Future exploration of such variables may highlight differential mechanisms for the modelling of behaviours related to food.

Another limitation of the study relates to potential selection bias. The ALSPAC cohort is now a three-generational study, comprising “G0,” i.e., the cohort of original pregnant women, the biological father and other carers/partners, “G1,” i.e., the cohort of index children, and “G2,” i.e., the cohort of offspring of the index children, from which our study sample was drawn. The G0 mothers are overall from somewhat higher socio-economic backgrounds compared to the general population, whilst the G1 participants who enrolled their children in G2 are more engaged with the ALSPAC study and more educated compared to those who did not participate in the study (37). Future research should examine this association with a larger sample, disaggregate the association by variation in ED diagnoses, and determine if these processes relate to child outcomes later in development, as we do not know that these early differences in attention are associated with ED behaviour outcomes. If differences in attentional patterns during feeding interactions confer risk, they could become sensible, modifiable targets for intervention. Intervention could include “light touch” strategies, such as psychoeducation knowledge exchange regarding the role of non-verbal behaviours/communications during feeding practices and psychoeducation about the relevance of attending to such aspects of caregiver–child interactions. The findings could also be used to develop interventions, for example, utilising video feedback and psychoeducation. Interventions could focus on supporting mothers to guide infant attention to non-food-related activities or stimuli in attempts to minimise any impact on the infant-to-mother and mother-to-infant relationships and/or the infant’s own relationship with food. Other work could include interventions to support the development of flexible, responsive interpersonal strategies to support the feeding interactions.

## Data Availability

The raw data supporting the conclusions of this article will be made available by the authors, without undue reservation.
