# Correction: Mothers’ eating disorder history and mother and infant attention to food during infant meal times: a candidate for intergenerational transmission of eating disorder behaviours

**DOI:** 10.3389/frcha.2026.1794833

**Published:** 2026-02-19

**Authors:** Fay Huntley, Rebecca M. Pearson, Ilaria Costantini, Marc H. Bornstein, Amy Campbell, Miguel Cordero, Nicky Wright

**Affiliations:** 1 School of Health in Social Science, Clinical Psychology, University of Edinburgh, Scotland, United Kingdom; 2 School of Psychology, Faculty of Health and Education, Manchester Metropolitan University, Manchester, United Kingdom; 3 Population Health Sciences, University of Bristol Medical School, Bristol, United Kingdom; 4 Division of Psychiatry, University College London, London, United Kingdom; 5 Eunice Kennedy Shriver National Institute of Child Health and Human Development, Bethesda, MD, United States; 6 Institute for Fiscal Studies, London, United Kingdom; 7 MRC Integrative Epidemiology Unit, University of Bristol, Bristol, United Kingdom; 8 School of Psychological Science, University of Bristol, Bristol, United Kingdom; 9 Centre for Academic Mental Health, University of Bristol, Bristol, United Kingdom; 10 Instituto de Ciencias e Innovación en Medicina, Universidad del Desarrollo Facultad de Medicina Clínica Alemana, Las Condes, Chile

**Keywords:** mothers, infants, eating disorder, attention, food, interactions

**Author name**

**Please also check that the initials used in the Author Contributions section or elsewhere in the article are correct.**

Misspelled

Author Ilaria Costantini was erroneously spelled as Ilaria Constantini. It should be Ilaria Costantini.

**Author contributions**

The author contributions statement was erroneously given as:

FH: Writing – original draft, Writing – review & editing.

RP: Software, Writing – review & editing, Methodology, Conceptualization, Investigation, Funding acquisition.

IC: Methodology, Writing – review & editing.

MB: Conceptualization, Writing – review & editing.

AC: Writing – review & editing.

MC: Writing – review & editing.

NW: Conceptualization, Writing – review & editing, Writing – original draft, Formal analysis, Methodology.

**The correct author contributions statement is:**

FH: Writing – Original draft, Writing – review & editing, Conceptualization.

RP: Writing – Original draft, Writing – review & editing, Conceptualization, Investigation, Software, Methodology, Supervision, Formal analysis, Project administration, Funding acquisition, Resources.

IC: Writing – Original draft, Writing – review & editing, Investigation, Software, Data curation, Resources.

MB: Writing – Original draft, Writing – review & editing, Supervision.

AC: Writing – Original draft, Writing – review & editing.

MC: Writing – Original draft, Writing – review & editing, Investigation, Software, Data curation, Resources.

NW: Writing – Original draft, Writing – review & editing, Conceptualization, Methodology, Supervision.

**Missing citation**

Wrong

The reference for: Costantini I, Cordero M, Campbell A, Burgess RJ, Glen K, Moraitopoulou G, et al. Mental Health Intergenerational Transmission (MHINT) Process Manual. Moscow: OSF (2021). doi: 10.31219/osf.io/s6n4h was erroneously written as: 43. Constantinou A. The Bayesys User Manual. London: Bayesian Research Lab (2019). p. 154.

It should be: Costantini I, Cordero M, Campbell A, Burgess RJ, Glen K, Moraitopoulou G, et al. Mental Health Intergenerational Transmission (MHINT) Process Manual. Moscow: OSF (2021). doi: 10.31219/osf.io/s6n4h

**Incorrect funding**

Missing

The funding statement: The author(s) declare that financial support was received for the research and/or publication of this article. The UK Medical Research Council and Wellcome (Grant ref: 217065/Z/19/Z) and the University of Bristol provide core support for ALSPAC. This publication is the work of the authors and Dr Costantini and Prof Pearson will serve as guarantors for the contents of this paper. A comprehensive list of grants funding is available on the ALSPAC website (http://www.bristol.ac.uk/alspac/external/documents/grant-acknowledgements.pdf); This work is part of a project that has received funding from the European Research Council (ERC) under the European Union's Horizon 2020 research and innovation programme (Grant agreement No. 758813; MHINT) was erroneously written as: The author(s) declared that financial support was received for this work and/or its publication. The UK Medical Research Council and Wellcome (Grant ref: 217065/Z/19/Z) and the University of Bristol provide core support for ALSPAC. This publication is the work of the authors and Rebecca Pearson will serve as guarantor for the contents of the article. This work is part of a project that has received funding from the Wellcome Trust (Grant ref: 084632/Z/08/Z) and the UK Medical Research Council and Wellcome Trust (Grant ref: 102215/2/13/2). A comprehensive list of grants funding is available on the ALSPAC website (http://www.bristol.ac.uk/alspac/external/documents/grant-acknowledgements.pdf).

It should be: The author(s) declare that financial support was received for the research and/or publication of this article. The UK Medical Research Council and Wellcome (Grant ref: 217065/Z/19/Z) and the University of Bristol provide core support for ALSPAC. This publication is the work of the authors and Prof Pearson will serve as guarantor for the contents of this paper. A comprehensive list of grants funding is available on the ALSPAC website (http://www.bristol.ac.uk/alspac/external/documents/grant-acknowledgements.pdf); This work is part of a project that has received funding from the European Research Council (ERC) under the European Union's Horizon 2020 research and innovation programme (Grant agreement No. 758813; MHINT).

