# Supplementary material for: Mothers’ eating disorder history and mother and infant attention to food during infant meal times: a candidate for intergenerational transmission of eating disorder behaviours
Source: Front Child Adolesc Psychiatry. 2026 Jan 16;4:1699643. doi: 10.3389/frcha.2025.1699643 (PMC12855412; doi:10.3389/frcha.2025.1699643)

Supplementary materials

Figure S1: A Directed Acyclic Graph (DAG) identifying relevant confounding variables for analysis


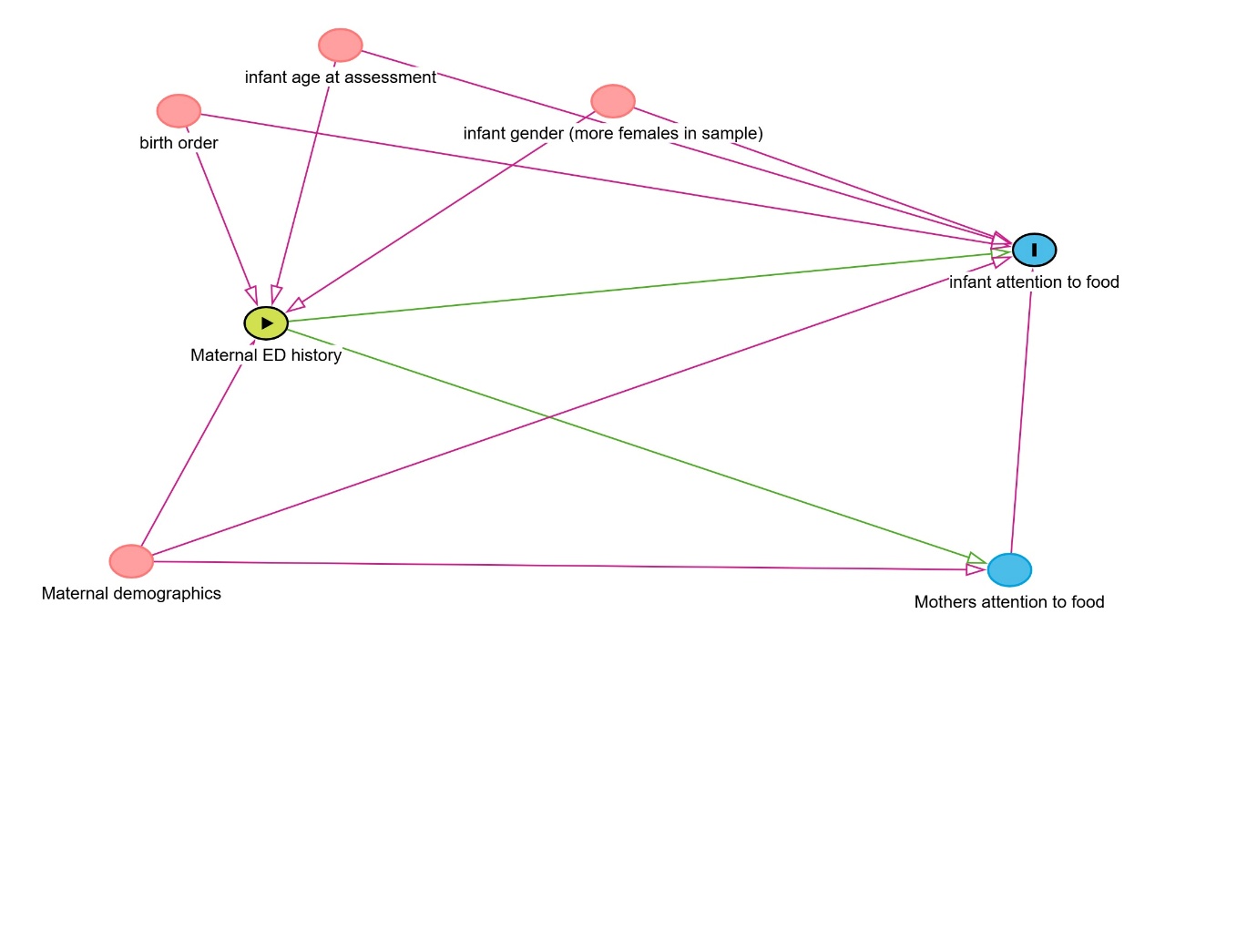


Note: This DAG was used to help map out the relationships between the variables of interest to identify which covariates to include in the model. Infant characteristics were included as they reflected who was more likely to contribute usable headcam data - particularly in relation to maternal ED history.

Figure S2: Scatterplot of association between mothers ED history behaviour and infant attention to food


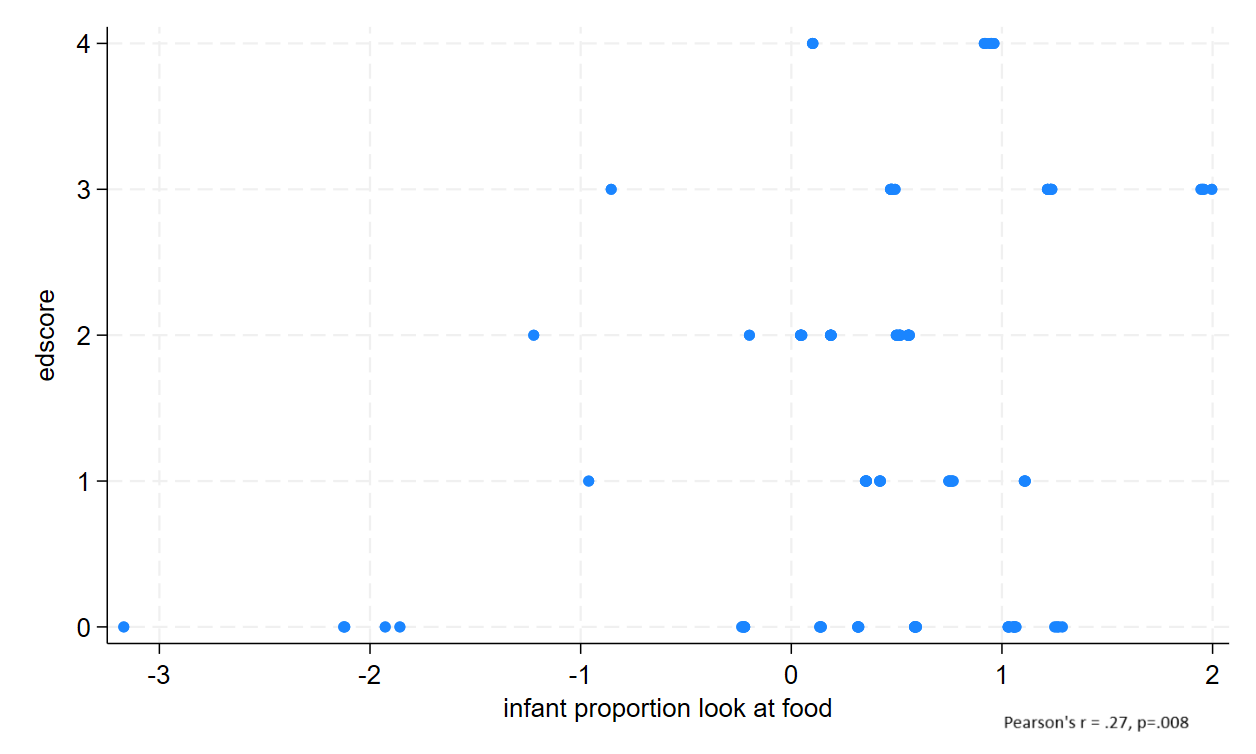


Figure S3: Scatterplot of association between mothers ED history behaviour and mother attention to food


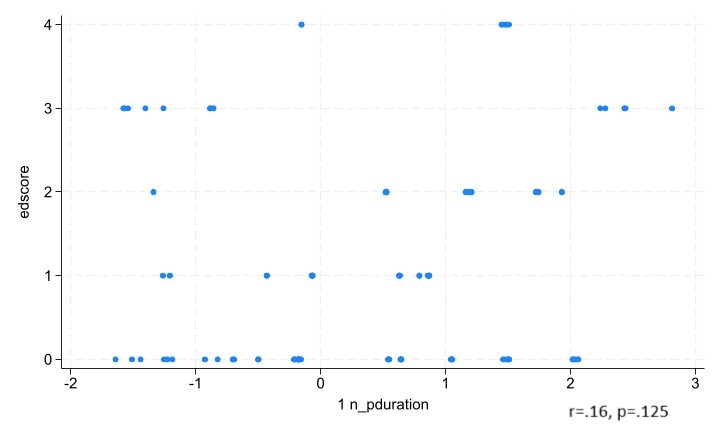

Supplement: Supplementary file 1 [file Table1.docx]
